# Seaweeds as a Functional Ingredient for a Healthy Diet

**DOI:** 10.3390/md18060301

**Published:** 2020-06-05

**Authors:** Rocío Peñalver, José M. Lorenzo, Gaspar Ros, Ryszard Amarowicz, Mirian Pateiro, Gema Nieto

**Affiliations:** 1 Department of Food Technology, Nutrition and Food Science, Veterinary Faculty University of Murcia, Campus Mare Nostrum, 30100 Espinardo, Spain; rocio.penalver@um.es (R.P.); gros@um.es (G.R.); gnieto@um.es (G.N.); 2 Centro Tecnológico de la Carne de Galicia, Parque Tecnológico de Galicia, 32900 San Cibrao das Viñas, Spain; mirianpateiro@ceteca.net; 3 Institute of Animal Reproduction and Food Research, Polish Academy of Sciences, 10-748 Olsztyn, Poland; r.amarowicz@pan.olsztyn.pl

**Keywords:** seaweeds, lipids, proteins, dietary fibre, polyphenols, functional food

## Abstract

Seaweeds have been used since ancient times as food, mainly by Asian countries, while in Western countries, their main application has been as gelling agents and colloids for the food, pharmaceuticals, and the cosmetic industry. Seaweeds are a good source of nutrients such as proteins, vitamins, minerals, and dietary fiber. Polyphenols, polysaccharides, and sterols, as well as other bioactive molecules, are mainly responsible for the healthy properties associated with seaweed. Antioxidant, anti-inflammatory, anti-cancer, and anti-diabetic properties are attributed to these compounds. If seaweeds are compared to terrestrial plants, they have a higher proportion of essential fatty acids as eicosapentaenoic (EPA) and docosahexaenoic (DHA) fatty acids. In addition, there are several secondary metabolites that are synthesized by algae such as terpenoids, oxylipins, phlorotannins, volatile hydrocarbons, and products of mixed biogenetic origin. Therefore, algae can be considered as a natural source of great interest, since they contain compounds with numerous biological activities and can be used as a functional ingredient in many technological applications to obtain functional foods.

## 1. Introduction

For several centuries, there has been a traditional use of seaweed as food in China, Japan, and Korea, as well as in some Latin American countries such as Mexico. The migration of the people from these countries around the world has meant that this custom has moved with them, so today, there are many more countries where seaweed consumption is not unusual. In recent years, there has been a strong movement in France to introduce seaweed into European cuisine, with some success, although it is still considered an exotic component of the menu. It has gained more acceptance in regions such as California and Hawaii, where Japanese communities are larger, and the taste for seaweed is spreading to the surrounding population as it is found in restaurant dishes and on supermarket menus. In fact, in Austria and Germany, seaweeds are being used to produce a highly prized bread—algenbrot, a blend of cereals whose composition is up to 3% seaweed. In Brittany, dulse and kombu are used to make the bara mor or “bread of the sea,” and minced seaweed in butter (beurre des algues) is used for cooking fish or spreading on bread to accompany shellfish [1].

On the east coast of the United States of America and Canada, some companies have begun to grow seaweed specifically for human consumption, and their markets are growing, both in those two countries and their exports to Japan. Cookbooks incorporating recipes with “sea vegetables” are on the market in many countries around the world. The growth of the world’s population and its concern for health, and the importance of the Earth’s limited and precious natural resources, has motivated the search for new foods that meet the needs of a growing population and at the same time provide some health benefit [2]. In fact, the demand for algae, whether for human consumption or for the processing of different industrial products, has increased in recent years, reaching a world production of 15.8 million tonnes in 2010 [3]. There are several algae used as human food, but some of the most recognized are *Porphyra*/*Pyropia* spp. (Nori), *Laminaria*/*Saccharina* spp. (Kombu), and *Undaria* spp. (Wakame). In some countries such as Japan and China, the cultivation of algae represents an industry that is expanding, since in Japan, the demand of algae for consumption is very high, with an average intake of 14.3 g/day per adult [4], reaching the recommended values of consumption of dietary fiber of 20–25 g/day [4].

In countries such as Japan, China, and Korea, approximately 66% of algae species have been used as a daily ingredient in their dishes for many years [5]. In fact, in the year 600 AD, Sze Teu wrote “algae are a delicious delicacy for the most representative guests, even for the king himself.” They are also consumed in some American countries such as Mexico, where has maintained the tradition of including algae in their diet. In contrast, in countries such as Spain and Portugal, where there are an extensive coasts and a great abundance of species, there is no tradition of including them in the diet. In recent years, however, this marine product is being revalued and managed to be introduced gradually into the diet, especially for vegans and vegetarians.

In Europe, substances exclusive to these plants, which have no synthetic equivalent, have been extracted from seaweeds. In this regard, seaweeds have been used for many years in the food and cosmetics industries [5,6,7]. In addition, seaweeds are an abundant, economical, and attractive resource for use as a food ingredient. They provide nutrients and bioactive compounds and have technological properties that make their incorporation viable. The concentration to be used must be correctly controlled, since sensory quality is not always favored, so it is an interesting challenge to include it in foods [8,9]. Finally, many studies have shown that the consumption of marine algae reduces the incidence of pathologies such as diabetes, obesity, cardiovascular diseases, cancer, etc. [10,11].

## 2. Definition of Seaweeds

Seaweeds are autotrophic organisms of simple structure with little or no cellular differentiation and complex tissues, so they are talophytes. They are classified taxonomically into three groups—Chlorophyta, Phaeophyceae, and Rhodophyta, corresponding to green, brown, and red algae, respectively [12]. Brown algae (phylum Ochrophyta, class Phaeophyceae) corresponds to a very large group of marine algae, although the exact number of species is not known. Its pigmentation varies from yellow to dark brown, hence its name. Within this group of algae, they are divided into two subgroups—kelps, such as *Laminaria hyperborea*, *Laminaria ochroleuca*, and *Saccorhiza polyschides*, which can measure several meters and form extensive submarine forests in the Western Cantabrians and Iberian Peninsula Atlantic coasts; and the fucales, such as *Fucus vesiculosus*, *Fucus serratus*, *Himanthalia elongata* (sea spaghetti), and *Ascophyllum nodosum*, algae that form very characteristic bands on the coastline. Red algae or Rhodophyta are the largest group of algae and are the most primitive, which are also found in different media. The species *Corallina officinalis*, *Tenarea tortuosa* (formerly *Lithophyllum tortuosum*), *Asparagopsis armata*, *Palmaria palmata*, and *Mastocarpus stellatus* are some examples. Finally, green algae or Chlorophyta have less presence than brown and red algae. Its pigmentation varies from greenish yellow to dark green. *Ulva lactuca*, known as sea lettuce, is the best known [13].

## 3. Nutritional Evaluation of Algae

The chemical composition of algae depends on the species, place of cultivation, atmospheric conditions, and harvesting period. From a nutritional point of view (Table 1), algae are an important source of proteins and lipids. In general, protein contents was higher in green and red seaweeds (10–47% of dry weight—DW) than those found in brown seaweeds (5–24% DW); in lipids (from 0.79% to 7.87% dry matter), ω-3 and ω-6 polyunsaturated fatty acids (PUFAs) constitute a significant part of the lipid profile of seaweeds [14,15,16,17,18,19,20].

On the other hand, dietary fiber is recognized today as an important element for healthy nutrition. However, there is no universal definition or analytical method that measures the food components that exert the physiological effects of fiber, but there is consensus that the definition should include the physiological role of dietary fiber [21]. Therefore, it can be said that dietary fiber consists of a series of compounds comprising a broad mixture of carbohydrates and polymers present in plants, including both oligosaccharides and polysaccharides, such as cellulose, hemicellulose, pectic substances, gums, resistant starch, and inulin, which may be associated with lignin and/or other non-carbohydrate components (polyphenols, waxes, saponins, cutins, and resistant protein) [22].

**Table 1:** Chemical composition of different algae (g/100 g dry weight—DW).

Seaweed	Protein	Lipids	Ashes	Ref.
**Chlorophyta**				
*Caulerpa lentillifera*	9.26 ± 0.03	1.57 ± 0.02	22.20 ± 0.27	[23]
*Ulva clathrata*	27.2 ± 1.1	2.2 ± 0.1	27.5 ± 0.2	[15]
*Ulva lactuca*	8.46 ± 0.01	7.87 ± 0.10	19.59 ± 0.51	[16]
**Rhodophyta**				
*Chondrus crispus*	27.2 ± 1.4	2.0 ± 0.1	21.1 ± 0.1	[24,25,26]
*Garateloupia turuturu*	22.9 ± 2.0	2.6 ± 0.1	18.5 ± 0.6	[14]
*Jania rubens*	11.28 ± 0.10	2.05 ± 0.09	44.03 ± 0.45	[27]
*Porphyra/Pyropia* spp.	26.6 ± 6.3	2.1 ± 1.2	20.6 ± 0.2	[19,24]
*Pterocladia capillacea* (formerly *Pterocladia capillacea*)	20.67 ± 0.03	2.19 ± 0.09	17.50 ± 0.28	[27]
**Phaeophyceae**				
*Ascophyllum nodosum*	8.70 ± 0.07	3.62 ± 0.17	30.89 ± 0.06	[28]
*Bifurcaria bifurcata*	8.92 ± 0.09	6.54 ± 0.27	31.68 ± 0.41	[28]
*Durvillaea antarctica*	11.6 ± 0.9	4.3 ± 0.6	25.7 ± 2.5	[17]
*Fucus vesiculosus*	12.99 ± 0.04	3.75 ± 0.20	20.71 ± 0.04	[28]
*Laminaria* spp.	6.3 ± 3.8	1.0 ± 0.3	37.6 ± 0.4	[19,24]
*Saccharina latissima*	25.70 ± 0.11	0.79 ± 0.07	34.78 ± 0.08	[18]
*Sargassum fusiforme*	10.9 ± 1.0	1.4 ± 0.1	-	[19]
*Undaria pinnatifida*	18.9 ± 9.8	4.5 ± 0.7	39.3 ± 0.2	[19,24]

Dietary fiber consists of two fractions (soluble and insoluble), and its properties are mainly determined by the proportion of these two fractions. Thus, soluble fiber is characterized by its ability to form viscous gels, in contact with water, in the intestinal tract. Insoluble fiber does not form gels in contact with water but is capable of retaining water in its structural matrix, producing an increase in fecal mass that accelerates intestinal transit. These differences in the behavior of the fibers in the intestinal transit result in different properties. Insoluble fiber is sparsely fermented and has a marked laxative and intestinal regulating effect, while soluble fiber is fermented in high proportion, and its main properties are related to the decrease of cholesterol and glucose in blood and the development of intestinal microbiota [29].

Algae are distinguished by the composition of the structural polysaccharides of the cell wall and reserve. Most of these polysaccharides can be considered as fiber, as they are not digested by human enzyme equipment, although some are degradable by enzymes produced by colonic bacteria [30]. The proportion of dietary fiber is considerable, ranging from 36% to 60% of its dry matter [31], with soluble dietary fiber being very high (approximately 55–70%) compared to terrestrial vegetables [5]. Within algae, the soluble fiber content is usually higher in red algae (15–22% dry weight) as in *Chondrus* and *Porphyra* (Nori) [8,32]. On the other hand, brown algae such as *Fucus* or *Laminaria*/*Saccharina*, have a higher insoluble fiber content (27–40% dry weight) [8,33]. Table 2 presents the dietary fiber values of different algae. Seaweeds have a high proportion of soluble fiber [34,35], with an average content of 24.5 g/100 g and 21.8 g/100 g for insoluble fiber. Finally, the ratio fiber soluble/fiber insoluble (S/I) is greater than the values observed in terrestrial vegetables.

**Table 2:** Dietary fiber content in different algae (g/100 g).

Seaweed	Soluble Fiber	Insoluble Fiber	Ref.
**Chlorophyta**			
*Caulerpa lentillifera*	17.21 ± 0.87	15.78 ± 1.20	[36]
*Enteromorpha* spp.	17.2	16.2	[37]
*Ulva* spp. (formerly *Enteromorpha* spp.)	21.9 ± 0.9	18.7 ± 2.1	[15]
*Ulva* spp. (formerly *Enteromorpha* spp.)	20.53 ± 0.28	34.37 ± 0.7	[16]
**Rhodophyta**			
*Chondrus crispus*	22.25 ± 0.99	12.04 ± 2.89	[32]
*Garateloupia turuturu*	48.1 ± 1.0	12.3 ± 1.2	[14]
*Porphyra/Pyropia* spp.	17.9	16.8	[37]
**Phaeophyceae**			
*Durvillaea antarctica*	27.7 ± 1.2	43.7 ± 0.3	[17]
*Himanthalia elongata*	23.63 ± 0.48	13.51 ± 0.45	[18]
*Himantalia elongata*	25.7	7.0	[37]
*Saccharina latissima*	17.12 ± 0.84	13.11 ± 0.56	[18]
*Sargassum fusiforme*	32.9	16.3	[37]
*Undaria pinnatifida*	30.0	5.3	[37]

Marine algae, because they live in an environment with a very high concentration of salts, need to accumulate solutes that allow to regulate the osmotic balance between their cells and the environment. Many ions such as sodium, chlorine, and potassium are involved in this process, but certain low molecular weight carbohydrates are also involved [38]. These include, for example, sucrose in green algae, alditols such as mannitol in brown and red algae [38], and hexitols such as digeneasides and fluorosides in red algae [39]. In addition, the main low molecular weight carbohydrate present in many species of brown algae, especially in *Laminaria* and *Ecklonia*, is mannitol. Mannitol content is <10% of dry weight in *Ascophyllyum nodosum* and *Laminaria hyperborea* species, although it is also subject to many seasonal variations, reaching maximum levels of up to 25% of dry weight in autumn [8]. Finally, seaweeds also contain a high concentration of carbohydrates such as structural, storage, and functional polysaccharides, with values between 20% and 70%. However, they are not a good source of carbohydrates in terms of bioavailability [5] due to the high proportion of soluble dietary fiber between 55–70%.

The algae acquire from the marine environment, in which they live, a great wealth of mineral elements, being known for its high content of minerals between 8–40% of the dry weight of the seaweed (Table 3). They are worth highlighting the great abundance of essential minerals such as sodium, calcium, magnesium, potassium, chloride, sulfate, phosphorus, and micronutrients such as iodine, iron, zinc, copper, selenium, molybdenum, fluoride, manganese, boron, nickel, cobalt, etc. [28]. However, the mineral composition may vary depending on the taxonomic group, geographical, seasonal and physiological variations [40], and even with the type of processing and mineralization method applied [41]. Algae are a primary source of iodine, providing the daily iodine requirement (150 μg/day) [5]. Because of their high mineral content, algae can be used as a dietary supplement to help achieve the recommended daily amounts of some macro minerals and trace elements.

Algae, besides being an important source of minerals, are an excellent source of vitamins [42]. Algae, depending on their habitat, season, and species, vary in vitamin content, but almost all spend a lot of time exposed to direct sunlight in a watery environment. As a result, algae contain many forms of antioxidants, including vitamins and protective pigments. Seaweeds contain both water- and fat-soluble vitamins [43,44]. In this regard, algae are an excellent source of vitamins A, B1, B12, C, D, and E; riboflavin; niacin; pantothenic acid; and folic acid [42]. Water-soluble vitamins such as vitamin C are present in large amounts in laver (*Porphyra umbilicalis*), sea spaghetti (*Himanthalia elongata*), *Crassiphycus changii* (formerly *Gracilaria changii*) [45,46], and brown seaweed *Ecklonia arborea* (formerly *Eisenia arborea*) [47].

Algae also are a good source of B-group vitamins (particularly B1 and B12), as well as the lipophilic vitamin A (derived from β-carotene) and vitamin E (tocopherols) [48,49]. Finally, seaweed foods offer one of the few vegetarian alternatives for cobalamin (vitamin B12) in the diet. Cobalamin is not required or synthesized by higher plants [50], so fruits and vegetables are poor sources of vitamin B12, which explains why vitamin B12 deficiency is common among people following strict vegetarian or vegan diets [51,52,53].

**Table 3:** Mineral content in different algae (mg/100 g DW).

	Macro-Minerals					Micro-Minerals				
Seaweed	Ca	K	Mg	Na	P	Fe	Mn	Zn	Cu	Ref.
**Chlorophyta**										
*Caulerpa lentillifera*	1874.7	1142.7	1028.6	8917.5	-	21.37	-	3.51	0.11	[36]
*Ulva rigida*	524.5	1561.0	2094.1	1595.0	210.0	283.0	1.60	0.60	0.50	[54]
**Rhodophyta**										
*Chondrus crispus*	420.0	3184.0	732.0	4270.0	-	3.97	1.32	7.14	<0.50	[24]
*Ellisolandia elongata* (formerly *Corallina mediterranea*)	45,075.2	759.3	4977.4	2457.7	-	27.70	6.27	3.02	0.69	[55]
*Jania rubens*	42,344.0	327.5	2986.6	2086.2	-	47.50	9.53	2.63	0.36	[55]
*Palmaria palmata*	1000.0	2700.0	200.0	1100.0	500.0	31.56	3.59	2.85	0.56	[56]
*Porphyra umbilicalis*	687.0	1407.0	283.3	1173.0	0.025	18.20	2.72	4.23	-	[57]
*Pyropia tenera* (formerly *Porphyra tenera*)	390.0	3500.0	565.0	3627.0	-	10.30	2.72	2.21	<0.50	[24]
*Pterocladiella capillacea* (formerly *Pterocladia capillacea*)	6105.0	1495.0	770.9	2949.5	-	22.70	3.33	4.21	0.43	[55]
**Phaeophyceae**										
*Alaria esculenta*	900.0	4400.0	700.0	3900.0	400.0	2.60	0.35	2.98	2.13	[56]
*Ascophyllum nodosum*	984.7	3781.4	867.8	4575.7	-	13.34	1.96	-	-	[28]
*Bifurcaria bifurcata*	996.4	9316.3	528.0	1836.8	169.5	-	-	-	-	[28]
*Fucus vesiculosus*	938.0	4322.0	994.0	5469.0	-	4.20	5.50	3.71	<0.50	[24]
*Himanthalia elongata*	909.0	6739.0	826.6	3700.0	0.015	1.81	4.09	3.77	-	[57]
*Laminaria digitata*	1005.0	11,579.0	659.0	3818.0	-	3.29	<0.50	1.77	<0.50	[24]
*Undaria pinnatifida*	931.0	8699.0	1181.0	7064.0	-	7.56	0.87	1.74	<0.50	[24]

The protein content of algae varies greatly between large groups of algae (brown, red and green). In brown algae, the protein content is generally low (5–24% of dry weight), while red and green algae have a higher protein content (10–47% of dry weight) [58]. As with other nutritional components of algae, the content of proteins, peptides, and amino acids is influenced by various factors, especially seasonal variation [8]. For example, brown algae *Saccharina* and *Laminaria* displayed the maximum protein content during the months of February to May [8]. A similar variation has been found in red algae species, with a maximum in summer and a considerable reduction during winter [58]. In general, algae proteins are rich in glycine, arginine, alanine, and glutamic acid; they contain essential amino acids at levels comparable to the requirements indicated by FAO/WHO. Their limiting amino acids are lysine and cystine [5,42]. Other amino acids present in algae are taurine, laminin, kainoids, kainic and domoic acids, and some mycosporin-type amino acids [59,60]. Taurine, in humans, participates in many physiological processes such as immunomodulation, membrane stabilization, ocular development, and the nervous system [61]. In addition, kainic and domoic acids are involved in the regulation of neurophysiological processes [62].

On the other hand, some studies have shown that phycobiliproteins extracted from red algae (phycoerythrin) could be beneficial in the prevention or treatment of neurodegenerative diseases caused by oxidative stress (Alzheimer and Parkinson’s) due to their antioxidant effects [63]. The lectins found in *Bryothamnion* spp. (Rhodophyta) show an inhibitory effect on the growth of strains of *Steptococcus* spp.; therefore, they can be used as bactericidal compounds [64]. Among the peptides found in algae, those with 2–20 amino acids abound. They can be linear, cyclic, depsipetides, or peptides with one or more amide bonds replaced by ester-kahalalides- bonds, dipeptides (carnosine, almazole D), tripeptides (glutathione), pentapetides (galaximide), hexapeptides, oligopeptides, and phycobiliproteins [65]. These isolated peptides are characterized by having antioxidant, antitumor, antiviral, antimicrobial, antihypertensive, anticoagulatory, and immunostimulatory activities [66]. In particular, the kahalalides P and Q present in green algae possess cytotoxic action on the HL-60 cell line, while the kahalalide F, isolated from *Bryopsis* spp. (Chlorophyta), reduces the density of non-metastatic prostate tumor cells [62]. The most abundant amino acids are lectins, phycobiliproteins, agglutinins, and glycoproteins [60].

## 4. Bioactive Compounds in Algae

Apart from their nutritional components, algae contain bioactive compounds with high antioxidant capacity, such as carotenoids and polyphenols [17,67,68,69,70,71,72]. The natural pigments of algae have been studied finding antioxidant, anticancer, anti-inflammatory (mainly based on modulating macrophage function) activity, among others [73]. Among the natural algae pigments stands out fucoxanthin, a carotenoid that is available in different species of brown algae [74]. In this regard, several authors [75,76,77,78,79,80] have shown that fucoxanthin from different types of algae have an antioxidant, anticancer, anti-inflammatory, anti-obesity, neuroprotective, photoprotective, and osteoporosis preventive effects [72,75,76,77,78,79,80].

Polyphenols are a minority component of algae (Table 4). Green and red algae contain low concentrations of polyphenols (<1% dry weight) compared to brown algae [32,40] and can reach up to 14% dry weight in *Ascophyllum* and *Fucus* genera [8]. Phlorotannins are the most widely described polyphenols of brown algae, especially in species of the genus *Ecklonia* [8,71], and are formed from phloroglucinol (1,3,5-trihydroxybenzene) oligomeric structures [81]. Phlorotannins can be found at concentrations of 20–250 mg/g dry weight in *Ascophyllum nodosum*, *Fucus vesiculosus*, *Sargassum spinuligerum*, and *Cystophora retroflexa* [82]. In addition to phlorotannins, other polyphenols have been described, such as fucol and its derivatives, flavonoids, and derivatives such as catechin and epicatechin [8].

**Table 4:** Polyphenols content in different algae.

Seaweed	Total Polyphenols	Ref.
**Chlorophyta**		
*Ulva lactuca*	2.86 ± 0.04 (mg GAE/100 g DW)	[83]
**Rhodophyta**		
*Ellisolandia elongata* (formerly *Corallina mediterranea*)	37 (mg GAE/100 g extract)	[55]
*Crassiphycus birdiae*	1.06 ± 0.07 (mg GAE/100 g extract)	[69]
*Jania rubens*	56 (mg GAE/100 g extract)	[55]
*Porphyra umbilicalis*	5.53 (g GAE/100 g DW)	[57]
*Pterocladiella capillacea* (formerly *Pterocladia capillacea*)	93 (mg GAE/100 g extract)	[55]
**Phaeophyceae**		
*Alaria esculenta*	2.80 ± 0.05 (mg GAE/100 g DW)	[83]
*Ascophyllum nodosum*	0.96 ± 0.03 g PGE/100 g extract	[84]
*Bifurcaria bifurcata*	1.99 ± 0.23 g PGE/100 g extract	[84]
*Fucus vesiculosus*	1.15 ± 0.02 g PGE/100 g extract	[84]
*Halopteris scoparia*	328.7 ± 2.87 (mg GAE/100 g DW)	[85]
*Himanthalia elongata*	23.47 (g GAE/100 g DW)	[57]
*Saccharina latissima*	11.1 mg GAE/g DW	[56]
*Turbinaria conoides*	0.86 (mg GAE E/100 g DW)	[67]
*Undaria pinnatifida*	4.46 (g GAE/100 g DW)	[57]

DW–dry weight; GAE–gallic acid equivalents; PGE–Phloroglucinol equivalents.

The lipid algae content is low (1–5%), with neutral lipids and glycolipids being the most abundant. The proportion of essential fatty acids in algae is higher than in terrestrial plants, because they synthesize long chain polyunsaturated fatty acids, highlighting the eicosapentaenoic acid (EPA) and docosahexaenoic acid (DHA) that belong to the family of fatty acids ω-3 [86]. In general, red algae have high EPA, palmitic acid, oleic acid, and arachidonic acid contents compared to brown algae, which contain high concentrations of oleic acid, linoleic acid and α-linolenic acid but low EPA. Green algae have in greater quantity linoleic acid and α-linolenic, palmitic, oleic and DHA [87,88]. Both red and brown algae are a source of omega-3 and omega-6 fatty acids [40,89] Table 5 presents the proportion of EPA and DHA in algae and the relationship ω-6:ω-3.

## 5. Biological Properties of Algae

**Figure 1:**
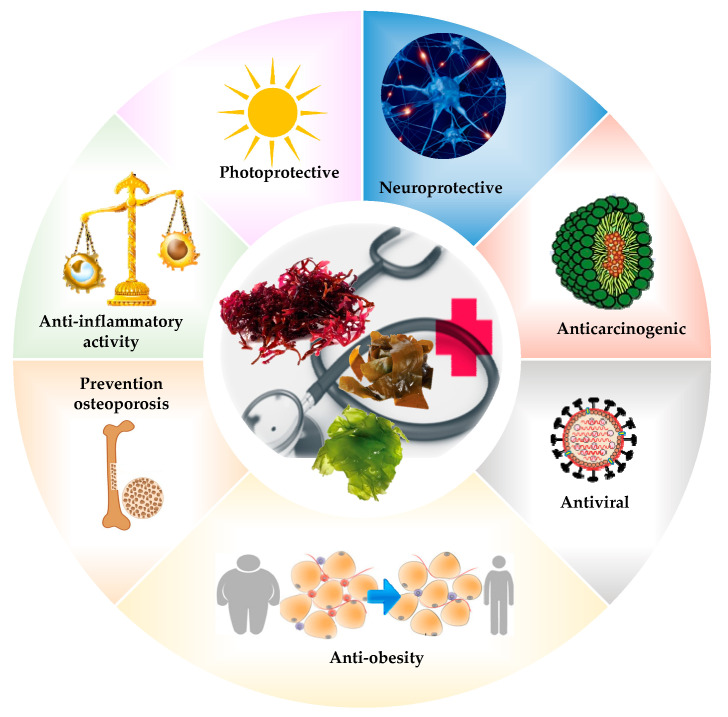
Biological properties of seaweeds.

The bioactive compounds present in seaweeds give them properties associated with the prevention and treatment of diseases (Figure 1), such as antidiabetic, antihypertensive, anti-inflammatory (based mainly on the modulation of macrophage function), antimicrobial, antitumor, antivirus, fat-lowering, and neuroprotective agents [73,90,91]. Primary and secondary metabolites can be also implicated in these applications. Primary metabolites are proteins, polysaccharides, and lipids involved in physiological functions. Among them, polysaccharides and fibers are the main compounds that display positive effects on chronic diseases such as cancer, cardiovascular diseases, diabetes, and obesity. On the other hand, secondary metabolites are minor molecules, such as phenolic compounds, halogenated compounds, sterols, terpenes, and small peptides, which are the result of stressful situations on seaweed tissues. Between them, exposure to ultraviolet radiation, changes in temperature and salinity, or environmental pollutants should be highlighted [92].

### 5.1. Antibiotics, Antifungals, and Antiviral Activity

Today, microbial resistance to several antibiotics makes it necessary to look for new antimicrobial agents in natural compounds [93]. These compounds would lead to more effective and less toxic compounds [94]. Recently, chemical investigations carried out on macroalgae have shown that these organisms produce a wide variety of biologically active secondary metabolites with unique molecular structures not found in other organisms. The three groups of seaweeds (Chlorophyta or green algae, Rhodophyta or red algae, and Phaeophyceae or brown algae) have exhibit pharmacological activity, inhibiting the growth of certain bacteria, viruses, and fungi [95]. In this way, studies conducted with brown algae *Ascophyllum nodosum*, *Laminaria* spp., and *Sargassum* spp. confirmed that could be used to combat infectious diseases caused by pathogenic bacteria [96].

There are numerous studies based mainly on the evaluation of the activity of the algae extracts obtained from different parts of the world against a fairly large spectrum of pathogenic bacteria (Gram-positive and Gram-negative), viruses, and fungi. Therefore, seaweeds could be used as promising antimicrobial agents in medicine. In this way, methanolic extracts from *Ulva lactuca* displayed good properties against pathogenic bacteria as Gram positive (*Bacillus subtilis*, *Staphylococcus aureus*, *Staphylococcus epidermidis*, and *Bacillus* spp.) and negative (*Escherichia coli*, *Klebsiella* spp., *Pseudomonas aeruginosa*, and *Salmonella typhi*) [97].

In addition, antiviral activities against human immunodeficiency virus (HIV), Herpes simplex virus (HSV), and respiratory syncytial virus (RSV) were displayed by seaweeds. Sulfated polysaccharides such as carrageenans, fucoidans, and sulfated rhamnogalactans are associated with their inhibitory properties against to cell damage produced by HIV-1 [98,99,100,101]. This is the case of species as *Agardhiella subulata* (formerly *Agardhiella tenera*), *Nothogenia fastigiata* spp., or *Ulva lactuca*. In addition, the chemical characterization of any of the active compounds showed the seasonal variation in the activity of *Schizymenia dubyi* (Rhodophyta) extracts against the HIV-1 virus [102]. This macroalgae is a red algae that contains a high level of glucuronic acid, a polysaccharide called glucurono-galacto-sulfate that is responsible of antiviral activity [103].

### 5.2. Antioxidant Activity

Seaweed, in response to the highly oxidative conditions in which they live, have developed strong antioxidant defense systems. In the same way as photosynthetic organisms, algae are exposed to a combination of light and high oxygen concentrations, which allow the formation of free radicals and other strong oxidizing agents [104]. However, the absence of oxidative damage to the thylakoid membranes of its chloroplasts suggests that their cells have developed powerful protection mechanisms [105]. As a result, seaweeds contain in their chemical composition a wide range of bioactive compounds such as polyphenols, sulfated polysaccharides, unsaturated fats, peptides, and amino acids, which exhibit multiple antioxidant properties. Among them, the most significant are phlorotannins, fucoidans, and carotenoids [106,107,108].

Some seaweeds produce high amounts of polyphenolic secondary metabolites. Phlorotannins, which exhibit important antioxidant capacity related with their structure, can be used as potential substitute of synthetic antioxidants in the food industry [108]. These bioactive compounds, typically present in brown algae, have a high number of isomers due to the significant variation in branching positions between their phloroglucinol units (PGUs). The availability of hydroxyl groups in their structure and PGUs oligomerization seem to be responsible of the antioxidant activity of phlorotannins [109]. In addition, seaweeds also contain polyphenols with specific biological activity, which can affect gene expression [110,111]. Therefore, there is a great scientific interest in the properties of polyphenols related to the prevention of aging and cardiovascular and cancer diseases [112,113].

The polysaccharides present in brown algae such as fucoidans, laminarans and alginic acid show in vitro antioxidant activity [65] and can be considered as powerful potential antioxidants. The antioxidant activity of sulphated polysaccharides depends on several factors such as the degree of sulfation, molecular weight, type of sugar, and glycosidic bond [114,115]. Low-molecular-weight polysaccharides display higher antioxidant activity than those obtained at higher molecular weight [116] because these polysaccharides are incorporated more easily to cells and donate protons more efficiently than higher-molecular-weight polysaccharides [114]. Furthermore, a positive correlation was found between sulphate content and free radical scavenger activity in fucoidan fractions of brown algae *Saccharina japonica* (formerly *Laminaria japonica*) [117]. In addition, Sargachromanol E extracted from brown algae *Sargassum horneri* should be highlighted for its scavenging ability against ROS, protecting cell membranes from oxidative modification in UV-exposed human dermal fibroblasts [118].

Carotenoids are also efficient antioxidants present in seaweeds [108]. The most abundant carotenoids are xanthophyll and tocopherols. Xanthophylls are efficient quenchers of singlet oxygen, being fucoxanthin the most prominent, while tocopherols are widely used in the food industry due to their efficient radical scavenging activity.

### 5.3. Anticoagulant Activity

The anticoagulant capacity of sulphated polysaccharides from algae has been one of the most studied properties, in order to find a substitute of natural origin for heparin. The anticoagulant capacity of the polysaccharides of algae will depend on many factors such as weight molecular, the composition of sugars, degree and distribution of sulphate groups in the molecule, etc. [100]. Several sulphated polysaccharides with capacity anticoagulant have been isolated and characterize from algae [114]. Mainly, two types of sulphated polysaccharides have been identified, the sulphated galactans or carrageenans of red algae [114], and sulphated fucoidans from algae browns [119,120].

The relationship between structure and anticoagulant capacity of some sulphated polysaccharides from algae have been studied by several groups [119,121,122], noting that the presence of sulphate groups appears to be a determining factor in this capacity. The results obtained so far demonstrate that algae polysaccharides may be an alternative to heparin due to its potential use as natural anticoagulant substances in the pharmaceutical industry [100,114].

### 5.4. Anticancer Activity

Cancer is one of the main diseases detected in developed countries [123]. Several treatments have been development to cure this disease; however, these therapies have many secondary effects. In this situation, new therapies need to be found to help alleviate these side effects. The polysaccharides obtained from seaweeds could be an alternative, since they have shown anticancer activity against some types of cancer [124]. Their effect on tumor cells is due to several modes of action, such as cell cycle arrest, depolarization of mitochondrial membrane, DNA damage, and nitric oxide production [123].

Algae produce a variety of chemically active metabolites to protect themselves from other settlement agencies [125]. Preliminary studies have indicated that some antioxidants, particularly β-carotene, may be beneficial in the treatment of precancerous conditions such as oral leukoplakia, a possible precursor of oral cancer [126].

Polysaccharides play an important role in immune regulation, and some of them are well-recognized anti-inflammatory molecules. Promising applications are linked to alginate, carrageenans, laminaran, peptides, phlorotannins, or porphyran. Phlorotannins are important in cancer chemoprevention. In this way, phlorotannins extracted from the brown algae *Ecklonia cava* protect cells from radiation-induced injury and from oxidative stress [127]. Fucoidan and fucoxanthin are important seaweed metabolites known for their antitumor effects and are used as potential chemotherapeutic or chemopreventive agents. Fucoidan is an L-fucose-rich sulphated polysaccharide extracted from brown seaweeds. Some studies reported that fucoidan exhibits several anticancer effects, including induction of apoptosis and inhibition of tumor-induced angiogenesis [128,129]. Effects on hepatocarcinoma and melanoma cancer have been reported, as well as in bladder, breast, colon, liver, lung, and prostate cells [130,131].

Insoluble fiber is characterized by its porosity and low density, which allows it to increase fecal mass and decrease intestinal transit [22]. In addition, the polysaccharides that compose the fiber can be potentially beneficial in gastrointestinal health, contributing to intestinal transit with a positive influence in the prevention of colon cancer [30].

Moreover, the crude extracts of brown seaweeds also displayed anticancer activity. This is the case of *Sargassum wightii*, which avoids the advance of breast cancer cell lines with a proportional dose effect [132]. Antiproliferative effect were also observed in *Bifurcaria bifurcata*, *Carpodesmia tamariscifolia* (formerly *Cystoseira tamariscifolia*), *Desmarestia ligulata*, *Dictyota dichotoma*, and *Halidrys siliquosa* crude extracts [133].

### 5.5. Neuroprotective Activity

Several neuroprotective effects have been attributed to seaweeds, especially to brown species [134,135]. Parkinson’s disease is one of them, a common neurodegenerative disease associated with movement difficulties and characterized by the accumulation of Lewy bodies, loss of dopaminergic neurons in the substantia nigra of pars compacta (SNpc), and depletion of the neurotransmitter dopamine. This illness does not have cure, and the mechanisms involved are not known with certainty. Therefore, it is necessary to look for new prevention and control therapies.

In addition to its well-known properties for the design of functional products, the red seaweed *Cistus crispus* showed, through a transgenic *Caenorhabditis elegans* (Nematoda) PD model, very good results, since it reduced the accumulation of α-synulein and protected nematodes from the neurotoxic 6-hydroxydopamine (6-OHDA) and induced dopaminergic neurodegeneration. These promising results could allow its use in the pharmaceutical field for the design of new anti-neurodegenerative drugs [136].

Sevevirathne et al. [137] demonstrated the anti-Alzheimer’s, anti-inflammatory, and antioxidant properties of the enzymatic hydrolysates from brown seaweed *Saccharina japonica* (formerly *Laminaria japonica*). This antioxidant activity depended on the proteases and carbohydrases used. However, acetylcholinesterase inhibitory activity was higher in the hydrolysates obtained with Flavourzyme (90%) and Celluclast (60%). In addition, hydrolysates displayed a high cell viability, which allow its use in pharmaceutical field.

The bioactive compounds present in seaweeds play an important role in neurodegenerative molecules. This is the case of polysaccharides. The phlorotannins, characteristic of brown seaweeds, have potential as therapeutics for human health [138]. The use of pre-treatment, type of solvent, drying temperature, particle size, temperature, extraction time, and solid/liquid ratio upon extraction all affect its quality and, therefore, its effectiveness. Phloroeckol and a tetrameric phloroglucinol of *Macrocystis pyrifera* (Phaeophyceae) would be responsible for its antidiabetic and anti-alzheimer’s effect, and antiallergic effect, respectively. Cha et al. [139] noticed the potential use of the edible brown seaweed *Ecklonia cava* as therapeutic agent for the prevention of Parkinson’s disease. Its anti-neurodegenerative activity could be linked with the antioxidant activity of dieckol in dopaminergic neuronal cells. The vulnerability of these cells to oxidative stress gives rise to intracellular toxic events, which result in protein aggregation, leading to cell death and the appearance of Parkinson’s disease. This polyphenol isolated from the seaweed prevents α-synuclein aggregation.

Natural algae pigments present in seaweeds also have neuroprotective effects. Fucoxanthin obtained from *Undaria pinnatifida* (Phaeophyceae) displayed a promising effect, since can attenuate neuronal cell damage in cortical neurons under hypoxia and re-oxygenation [140]. This fact could be due to the presence of allenic bond and intramolecular oxygen atoms in its molecular structure [141]. Notable effects were found in the xanthophyll astaxanthin, such as the suppression of 6-OHDA-induced apoptosis, the inhibition of the production of inflammatory mediators, effects against H_2_O_2_-induced toxicity and against cerebral ischemia [134]. These effects could be due to the strong antioxidant properties that characterize this pigment, probably linked with the presence of many conjugated double bonds in its molecule.

### 5.6. Tissue Engineering

Tissue engineering investigates the repair of damaged or malfunctioning tissues or organs as a substitute for whole organ transplantation [142]. In addition to the effects discussed above, fucoidan-enriched seaweed extracts are used in osteoarthritis treatment. They contribute to mineral deposition in bones, increasing the activity of alkaline phosphatase and the level of osteocalcin [143]. Fucoidan obtained from *Undaria pinnatifida* displayed positive effects by reducing osteoarthritis symptoms [144].

Alginates also had a tissue-regeneration effect on several organ, bone, and cartilage defects. Barralet et al. [145] reported the use of alginate-based biocompatible hydrogels in the stem-cell transplantation. The use of the polysaccharide laminaran obtained from the brown algae *Saccharina longicruris* can accelerate the tissue-generation process [146]. Repairing effects were also reported by Bhadja et al. [147] in low-molecular-weight polysaccharides of *Betaphycus gelatinus* (formerly *Eucheuma gelatinum*), *Gracilariopsis lemaneiformis* (formerly *Gracilaria lemaneiformis*), *Pyropia yezoensis* (formerly *Porphyra yezoensis*) (Rhodophyta), *Saccharina japonica* (formerly *Laminaria japonica*), *Sargassum fusiforme*, and *Undaria pinnatifida* (Phaeophyceae) on kidney epithelial cells.

### 5.7. Other Activity

Algae have a high proportion of soluble fiber [34,35], which is characterized by its ability to increase viscosity, reduce glycemic response and plasma cholesterol [22]. The main products of fiber fermentation are short chain fatty acids (SCFA), mainly acetic, propionic, and butyric, which lead to a drop in pH and even to the stimulation of the growth of certain micro-organisms, modifying the bacterial metabolism in the colon [8], known as the prebiotic effect. In addition, SCFA may have a beneficial effect on cholesterol metabolism [148].

The biological effect of EPA and DHA is very extensive and varied, involving lipoproteins, blood pressure, cardiac function, endothelial function, vascular reactivity, and cardiac physiology, as well as anti-inflammatory and antiplatelet effects [86,149]. They have positive effects on reducing the risk of cardiovascular diseases [150], triglyceride levels [151], and are necessary during pregnancy and lactation for the development of the central nervous system and retina of the infant. Moreover, their consumption displayed positive effects against postpartum and bipolar depression [152,153].

## 6. Inclusion of Seaweed in Food Products

**Figure 2:**
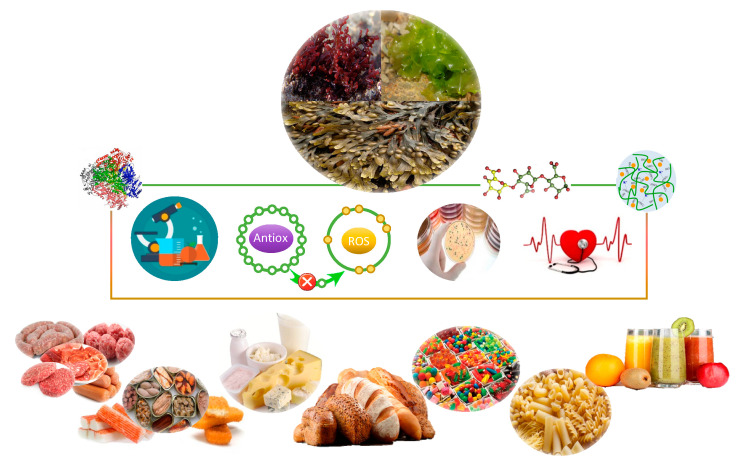
Functional and technological applications of seaweeds and their extracts in food products.

Seaweeds are renewable sources of high-added value compounds, which has attracted the interest of the food industries [154]. Their use in bakery, dairy, fish, meat, or vegetable-based products allow for the development of new functional food products (Figure 2), fortifying their nutritional composition, their quality, and their health-related beneficial properties [155]. Furthermore, macroalgae can be also used by their technological properties, since they contain in their composition phycocolloids, such as agar, alginates, and carrageenan, which are highly valued for their gelling, thickening, and stabilizing properties [156,157]. It is estimated that these compounds involve the 39% of the world production of colloids [158]. The contents of bioactive and technological compounds depend on the species. Thus, brown algae stands out for its contents in alginic acid, fucoidan, and laminarin present in *Macrocystis* spp., *Laminaria* spp., and *Ascophyllum* spp.; green algae has significant contents of ulvans; and red algae are rich in agar (*Gelidium* spp.), carrageenans (*Chondrus* spp.), floridean starch, porphyran, water-soluble sulfated galactan, and xylans [159].

Agar, known as “vegetable gelatin,” are present in *Gracilaria* and *Gelidium* especies [160]. It is generally recognized as safe (GRAS), which allows their use in food products as safer additive. In fact, it is the first hydrocolloid with European registration number E406 [26,161]. This ingredient, used in the production of jellies and fruit candies, forms a rigid gel in water at room temperature without the addition of potassium and calcium salts, even at low concentrations [18]. Moreover, is able to maintain its consistency even at high temperatures due to its melting point (85–95 °C), making it a highly valued product for food applications [26]. It is used in the formulation of some food products, such as canned meat products.

Alginate is an excellent stabilizing and thickening agent used in manufactured products due to its properties to chelate metal ions and form highly viscous solutions [162]. It is commonly used in desserts, drinks, ice cream, jelly, syrups, flavor sauces, fruit juices, bakery products, and milk shakes. This viscosity regulator serves as fat replacer ingredient, ensures smooth textures and improves the appearance, enhancing the overall quality of the products [163,164]. To this family of phycocolloids belong β-D-mannuronic acid units, α-L-guluronic acid units, and a third type with alternating β-D-mannuronic and α-L-guluronic acid unit polymers, available as safer additives with the European registration numbers E401 to E405 [157,161].

Carrageenan has many applications but binding water efficiently is probably what defines it as a hydrocolloid with excellent functional properties, improving the appearance and acceptability. The chemical forms found in seaweeds are κ- carrageenan and ι-carrageenan, which can form gels, and λ-carrageenan that is a thickening substance. Dairy and baking products are the main foods where it is used. In dairy products, such as cheese, chocolate milk, and cocoa, it has a very important role since it binds milk proteins, maintains milk solids in suspension, prevents fractionation of protein whey in cheese products, and enables crystallization in milk ice creams [157]. Carrageenans obtained from red algae of *Eucheuma* and *Kappaphycus* genera are used as fat replacers to produce healthier meat products with the aim of facilitating moisture retention and improving tenderness [165]. It has also been applied as a gas barrier on fresh cut fruit packaging, avoiding discolorations and maintaining texture during storage [166]. These hydrocolloids can also be used in bread, infant formula, jam, syrups, sauces, etc. [157,163]. The additive nomenclature applied to carrageenan is E407 [161].

Seaweeds have been added to meat products, such as burgers, frankfurters, pâtés, sausages, and steaks (Table 6). In these products, macroalgae have a double function: as antioxidants, preserving the quality of meat products; and as fat replacers, developing low-fat products [167]. In the first case, their powerful antioxidant properties are linked to the presence in their composition of bioactive compounds (alkaloids, carotenoids, polyphenols, terpenes, and tocopherols). This is the case of the brown seaweeds *Ascophyllum nodosum*, *Bifurcaria bifurcate*, and *Fucus vesiculosus* used in liver pâté with sufficient capacity to avoid the use of synthetic antioxidants, questioned for their harmful effects on health, thus providing lipid and protein stability to the product [168]. These results were confirmed in pork patties [169]. The high contents of phlorotannin in *Fucus vesiculosus* could explain its positive effects on oxidation stability (lipid and protein oxidation) and sensory attributes.

The antioxidant and antibacterial activities of the polysaccharides laminarin and fucoidan of *Laminaria digitata* were evaluated by Moroney et al. [170] in fresh and cooked minced pork patties. The oxidation processes produced lower redness values, which were dependent on the dose of algae used. In addition, the behavior of lipid oxidation was different in fresh and cooked samples. In fact, the higher dose used displayed a pro-oxidant effect, probably due to the contents of sodium, copper, and iron present in the extract. In contrast, cooked samples displayed an increase in the oxidation stability, which could be due to Maillard reaction products. These results were corroborated in further studies [171]. The results obtained for these polysaccharides separately demonstrated the higher antioxidant activity of fucoidan, which reduced lipid oxidation reactions, while laminarin did not contribute to the oxidative stability of fresh pork.

Positive effects of *Himanthalia elongata* (10–40%) was also noticed in beef patties [172]. The phenolic compounds present in this seaweed could be responsible for the oxidation and microbial stability of the products made with this seaweed. The scores obtained for sensory attributes (appearance, aroma, taste, and texture) confirmed its protective effect. In poultry products, positive effects were also shown for the use of seaweeds. The red seaweed *Kappaphycus alvarezii* was used in mechanically deboned chicken meat sausages as an antioxidant [173], reducing lipid oxidation and increasing redness values. Protection against oxidation was also displayed in cured turkey meat sausages manufactured with the brown algae *Treptacantha barbata* (formerly *Cystoseira barbata*) [174].

In low-fat meat products, seaweeds are emulsifiers that maintain fat retained and distributed in the product [161]. This strategy is motivated by the demands of consumers, who are increasingly concerned about their health, and by the recommendations of international organizations related to the decrease in the consumption of saturated fats and sodium contents [175]. Several fat replacers can be used in the manufacture of these products. These compounds depend on their nature, since protein-, lipid-, and carbohydrate-based replacers can be used [164]. The use of one or the other determines the characteristics of the final product [167].

K-carrageenan extracted from seaweeds was used as an emulsifier in low-fat reformulated meat products. Previous studies demonstrated that partial fat substitution with hydrocolloids can modify the texture and sensory characteristics of meat products. These effects were observed in beef sausages, where 70% of fat were substituted with κ- carrageenan. The texture changes obtained were dose-dependent, resulting in a decrease of hardness and chewiness and in an increase of springiness and gumminess parameters [176]. As mentioned previously, alginates are other hydrocolloids obtained from seaweeds. In this case, its application in low-fat ground pork patties increased cooking yield and resulted in a decline in flavor score as the concentration used increased [177]. Jellifying agents in the market, which contain sodium alginate in their composition, have been applied in burgers to replace animal fat by healthier vegetable oils [178,179].

**Table 6:** Effect of seaweeds addition on food properties.

Seaweed	Content	Product	Target	Outcome	Ref.
**Chlorophyta**					
*Caulerpa racemosa*	1.0%, 5.0%, and 10% substitution refined flour	Semi-sweet biscuits	Functional antioxidant	Increase water and oil absorption capacity of flour mix; Enhance nutritive and antioxidant value; Decrease sensory scores at high levels	[180]
*Cladophora* spp. *Ulva* spp.	2.5, 5.0 and 7.5% (based on wheat flour)	Bread	Nutrition	Increases in protein and fiber content; Slight changes in sensory and technological characteristics	[181]
*Ulva intestinalis*	Powder (2.77 g/kg) SP (0.5 g/kg)	Fish surimi	Functional and antioxidant effects	Maintain quality; Lower TBARs values over six months; Acceptable for juicy texture due to less cooking loss	[182]
*Ulva lactuca* *Ulva rigida*	1000 mg/kg	Pork patties	Natural antioxidants	Lower TBARs and metmyoglobin values than control	[183]
**Rhodophyta**					
*Crassiphycus birdiae* (formerly *Gracilaria birdiae*) *Gracilaria domingensis*	40%	Dairy dessert	Thickening agents	Enhance firmness; Good sensory acceptability; Maintain populations of *Bifidobacterium animalis* as probiotic	[156]
*Kappaphycus alvarezii*	2–8% powder	Dough and bread	Bread-making improver	Increase water absorption dough; Reduce stickiness properties; Higher firmness values	[184]
	0%, 2%, 4%, and 6% powder	Mechanically deboned chicken meat sausages	Natural antioxidant	Increased WHC and reduced water loss; Increase hardness and chewiness; Reduced lipid oxidation; Decreased lightness and increased redness	[173]
*Palmaria palmata*	4% protein hydrolysate	Bread	Increase health value	No changes in textural parameters and sensory scores; Retain renin inhibitory bioactivity	[185]
**Phaeophyceae**					
*Ascophyllum nodosum* *Fucus vesiculosus* *Bifurcaria bifurcata*	500 ppm	Pork liver pâté	Oxidative stability	Significant increase protein content; Best color parameters; Similar degree of protection against oxidation to synthetic antioxidant; Lower total volatile compounds	[168]
*Fucus vesiculosus*	0.5% and 1.0% acetone, ethanol, and water extracts	Granola bars fortified with fish oil-in-water emulsion	Antioxidant	Physical stability; Inhibit lipid oxidation; Affect physical microstructure of oil droplets, which were more spherical	[186]
	250, 500, and 1000 mg/kg	Pork patties	Natural antioxidants	Lower TBARs and carbonyl contents; No color enhancement during storage; No significant difference among batcher in sensory evaluation	[169]
*Himanthalia elongata*	5–15% of overall flour	Breadsticks	Functional product	Acceptable edible texture and color; Higher dietary fiber and phytochemical content	[187]
	3.3 g/100 g	Pork frankfurters	Technological application	Increased cooking loss; Reduced emulsion stability; More heterogeneous structure	[188]
	10–40% *w*/*w*	Beef patties	Functional antioxidant	Increased dietary fiber and TPC; Reduced cooking losses and hardness; Lower microbiological counts and lipid oxidation; Sensorially accepted by consumers	[172]
*Laminaria*	0.2%	Soft cheese	Functional purpose	Not degree quality; Slightly creamy; Spicy flavor	[189]
*Laminaria digitata* Laminarin-Fucoidan extract	0.01%, 0.1%, and 0.5%	Fresh minced and cooked pork	Antimicrobial antioxidant	Heat enhance antioxidant capacity; Pro-oxidant effect over time due to sodium, calcium, and iron contents of the extract; Lowest level can be incorporated without adverse effects	[170]
*Saccharina longicruris*	2% seaweed flakes	Camembert-type cheese	Antioxidant	Adequate development bioactivities during storage	[190]
*Sagassum wightii*	0%, 3.0%, and 5.0%	Tuna jerky	Functional ingredient	Induce positive effects on health; Improve nutritional, antioxidant, and microbial quality; Up to 3% not affect organoleptic quality	[191]
*Treptacantha barbata* (formerly *Cystoseira barbata*)	0.01%, 0.02%, and 0.04% (Fucoxanthin)	Cured turkey meat sausages	Natural antioxidant	Protection against oxidation: reduction of TBARs values, increased redness and yellowness values	[174]

WHC: water holding capacity; SP: sulphated polysaccharide.

Seaweeds were also used in fish and fish products by their functional properties but also for their protective effect during the product storage (Table 6). Brown seaweeds, and especially *Fucus vesiculosus*, are the most used in fish and oils [192]. Its positive effect on lipid oxidation was noticed in minced horse mackerel [193]. Moreover, this macroalgae could have beneficial effects on texture properties when it is used at levels of 1–2%, since the water-holding capacity associated with its fiber resulted in lower drip losses after thawing. On the contrary, Dellarosa et al. [194] did not find a noticeable effect of aqueous and ethanol extracts on lipid stability of fish cakes enriched with omega-3 PUFA. Nevertheless, no adverse effect was observed about their sensory scores. Another known brown seaweed is *Sargassum wightii*. Hanjabam et al. [191] suggested that this macroalgae could be used as a functional ingredient in ready-to-eat dried products such as tuna jerky. In addition to enriching the product with fiber, macro minerals, and trace elements, it improved antioxidant and microbial quality. Its incorporation in the product gave rise to brownish color, sponginess, and grassy flavor. However, the levels used (3–5%) resulted in a consumer acceptability similar to the samples without the seaweed.

The red seaweed *Kappaphycus alvarezii* (formerly *Eucheuma cottonii*) was tested at levels of 5%, 7.5%, 10%, 12.5%, and 15% in fish cutlets by Senthil et al. [195]. The water-holding capacity associated with its macroalgae and its powder affect the textural properties of the product. However, doses up to 10% can be used without adversely effects on appearance, texture, and acceptability. Furthermore, the antioxidant and biological activities associated with green seaweeds was confirmed by Jannat-Alipour et al. [182] in fish surimi. The use of *Ulva intestinalis* and its sulphated polysaccharide maintained the quality of the fish product, extending its shelf life.

Bakery products are the most consumed foods in the world [196]. However, many of these products are associated with unhealthy products, and contemporary consumers are much more aware and sensitized to take care of their health. In this situation, this sector is forced to reformulate their products. This reformulation must satisfy the nutritional demands of consumers without significantly affecting the organoleptic characteristics of traditional products. In fact, many industries usually enrich their products with protein or folic acid to nutritionally improve their products [197]. In this way, the addition of seaweeds could improve the image of bakery products, turning them into transmitters of their bioactive ingredients [196].

Bread, consumed daily in many countries, has been supplemented with skim milk powder, wheat, or soy proteins. However, these do not provide enough content of some essential amino acids, so it is necessary to look for other sources. Seaweed species have important contents of bioactive peptides, which could increase their contents in bread [198]. Red seaweed *Palmaria palmata* could be one of them, since this red seaweed displayed high protein contents between 9% and 25% and levels of lysine of 5.9 g/100 g of total amino acids. Levels of 4% of its protein hydrolysate would increase the health value of bread and retaining renin inhibitory bioactivity after baking without modifying textural parameters and sensory attributes [185].

Brown algae such as *Ascophyllum* spp. or *Laminaria* spp., green algae such as *Ulva lactuca*, and red algae such as *Pyropia tenera* (formerly *Porphyra tenera*) can also be used to supplement this product. The dose used is determinant for the quality of the resulted product, since inappropriate levels could result in harder products and changes in the color of the breadcrumbs, among others. Menezes et al. [181] evaluated the use of 2.5%, 5.0%, and 7.5% of *Ulva* spp. in conventional breads. The increase in protein and fiber content, and the slightly changes in sensory and technological characteristics, demonstrate the potential of green seaweeds. In the case of brown algae, Różyło et al. [199] showed that they should use levels up to 4% in the manufacture of gluten-free bread. The incorporation of red seaweed *Kappaphycus alvarezii* powder in bread at doses up to 8% could replace wheat flour without modify the quality of the final product, avoiding changes in color and the textural parameters of the dough [184].

Seaweeds have been also used in cereal-based products, such as semi-sweet biscuits, cereal bars, and breadsticks (Table 6). Semi-sweet biscuits are a healthier option of cookies, since they have less fat and sugar content. Green seaweed *Caulerpa racemosa* has been used in these biscuits to substitute refined flour at levels of 1.0%, 5.0%, and 10%. The results enhanced their nutritive (fiber, protein, and phenolic content) and antioxidant value in addition to increased water- and oil-absorption capacity of the flour–seaweed mix. In contrast, this supplementation decreased sensorial scores inversely to the level of *Caulerpa racemosa* [180]. *Himanthalia elongata* were used to develop functional products, since seaweeds are an important source of dietary fiber, and to enhance the phytochemical content of breadsticks at doses of 5–15% [187]. The obtained products had an attractive texture and color, which resulted in good sensory scores. *Fucus vesiculosus* was used as natural antioxidant in low-mixture foods such as granola bars [186]. The results showed that the brown algae resulted in an oxidative and physical stability of the product at levels of 0.5% and 1.0%. In addition, this incorporation resulted in more spherical oil droplets of the fish oil-in-water emulsion used to fortified cereal bars.

Seaweeds also improved the nutritional profile of flour and pasta [157]. In this way, *Undaria pinnatifida* (Phaeophyceae), known as Wakame, has been added to pasta in order to enhance its quality, since this algae present in its chemical composition appreciable amounts of fucoxanthin. It is one of the most important aquatic carotenoids with promising medicinal and nutritional properties. Its incorporation into pasta favored the interaction between the starch granules and the matrix protein [200]. The added doses should be adequate since they should not affect the sensory, technological, and textural properties of the product. Different levels of brown seaweed *Sargassum marginatum* (1.0%, 2.5%, and 5.0%) was also evaluated to increase the biofunctional, microstructure, and quality characteristics of pasta [201]. This seaweed, commonly used in alginate production, allowed to decrease cooking loss and enhance gluten network of pasta at levels up to 2.5%. Moreover, seaweed can be used to develop gluten-free foods, specially designed for the celiac population. Fradinho et al. [202] evaluated the use of brown seaweed *Laminaria ochroleuca* for gluten-free fresh pasta production. Its use resulted in a product with similar mechanical properties and higher fiber and mineral contents.

The use of seaweeds in dairy products is a promising strategy for the supplementation of fermented products (cheese, cottage cheese, cream, milk deserts, and yoghurt), improving their nutritional value, and enhancing their shelf-life (Table 6). In recent years, there have been many studies that have evaluated the supplementation of milk with macroalgae and their extracts [203,204]. In this way, O’Sullivan et al. [205] tested the brown algae species *Ascophyllum nodosum* and *Fucus vesiculosus* as functional ingredients in milk and noticed that the addition of seaweed extracts could improve milk quality and extend their shelf life. Other brown (*Himanthalia elongate*, *Laminaria ochroleuca*, and *Undaria pinnatifida*), green (*Ulva lactuca*), and red (*Chondrus crispus*, *Palmaria palmata*, and *Porphyra umbilicalis*) seaweed species were evaluated in milk by del Olmo et al. [206]. At the end of fermentation, higher probiotic contents were observed, probably due to the presence of inhibitory and stimulatory compounds in their extracts.

On the other hand, the great abundance of essential minerals and trace elements in seaweeds makes it possible to obtain healthy cheese and functional dairy products. Thus, the fortification of cheese with algae rich in calcium could avoid absorption problems generated by the immobilization of this mineral in casein. In addition, *Saccharina latissima* (formerly *Laminaria saccharina*) can be incorporated into cottage and fresh cheese to enrich these products and make them a source of iodine [189]. The functional use of this brown algae did not reduce the quality but resulted in spicy flavors. Furthermore, seaweeds could be also an alternative to reduce sodium contents in cheese [207]. This strategy would decrease salt contents associated with the development of cardiovascular and kidney diseases, complying with the recommendations made by WHO/FAO expert and consumer requirements [175]. *Palmaria palmate* and *Saccharina longicruris* flakes were used in Camembert-type cheese [190]. These seaweeds, which are rich in fiber, minerals, and protein, were added to replace the amount of salt and as a source of edible fiber. The ripening showed an adequate development of bioactivities.

Brown algae *Laminaria* were added to smoked cheese, milk deserts, and yoghurt. In this way, alginate oligosaccharides obtained from *Laminaria hyperborean* were used to supplement yoghurt with 2% (*w*/*v*) [208]. The results obtained displayed an antimicrobial effect of these oligosaccharides against some yeasts. However, as occur with other natural compounds, to achieve this preservative effect, a relatively high concentration should be used, which could change the sensory characteristics of yoghurt. Nuñez and Picon [203] evaluated the use of *Himanthalia elongate*, *Porphyra umbilicalis*, *Saccharina latissima*, *Ulva lactuca* and *Undaria pinnatifida* to supplement (0–1.0% dehydrated seaweed) yogurt and quark. The results obtained showed that these seaweeds can have sensory limitations due to the color and the untypical flavor intensity associated with these algae. However, these characteristics could be mitigated by selecting the right algae, since these parameters depend on the species.

In addition to its use to elaborate functional foods, aqueous extracts of seaweeds could be used for technological purposes in dairy products. This is the case of the Rhodophyta *Gracilaria domingensis*, mainly used for agar production. Results have supported its use as texture modifier in fermented milks, as well as its possible use as alternative to gelatine [156].

## 7. Conclusions

The nutritional quality of algae, due to its high content of protein, minerals, vitamins, dietary fiber, fatty acids, polysaccharides, and bioactive molecules with wide therapeutic potential, could further contribute to the improvement of the quality of human life and the increase of a balanced diet if consumed regularly. Different beneficial effects such as anticancer, antiviral, anticoagulant, hypocholesterolemic, and antioxidant have been demonstrated. In addition, algae have characteristic technological properties, which allows their incorporation in dairy, fish, meat, and pasta products, among others, maintaining or improving its sensorial, nutritional, and healthy quality.

## Figures and Tables

**Table 5:** Lipid content in different algae.

Seaweed	Lipids g/100 g	EPA (%)	DHA (%)	Ref.
**Chlorophyta**				
*Caulerpa lentillifera*	1.11 ± 0.05	0.86	-	[36]
*Codium fragile*	1.5 ± 0.0	2.10 ± 0.00	-	[49]
*Ulva lactuca*	1.27 ± 0.11	0.87 ± 0.16	0.8 ± 0.01	[17]
**Rhodophyta**				
*Agarophyton chilense*	1.3 ± 0.0	1.30 ± 0.01	-	[49]
*Porphyra/Pyropia* spp. (China)	1.0 ± 0.2	10.4 ± 7.46	-	[19,49]
**Phaeophyceae**				
*Ascophyllum nodosum*	3.62 ± 0.17	7.24 ± 0.08	-	[28]
*Bifurcaria bifurcata*	6.54 ± 0.27	4.09 ± 0.08	11.10 ± 1.13	[28]
*Durvillaea antarctica*	0.8 ± 0.1	4.95 ± 0.11	1.66 ± 0.02	[17]
*Fucus vesiculosus*	3.75 ± 0.20	9.94 ± 0.14	-	[28]
*Himanthalia elongata*	<1.5	7.45	-	[57]
*Laminaria* spp.	1.0 ± 0.3	16.2 ± 8.9	-	[19]
*Macrocystis pyrifera*	0.7 ± 0.1	0.47 ± 0.01	-	[49]
*Sargassum fusiforme*	1.4 ± 0.1	42.4 ± 11.9	-	[19]
*Undaria pinnatifida*	4.5 ± 0.7	13.2 ± 0.66	-	[19]
